# Retraction: Mendelian randomization based on immune cells in diabetic nephropathy

**DOI:** 10.3389/fendo.2025.1575593

**Published:** 2025-02-19

**Authors:** Frontiers Editorial Office

The journal retracts the August 06 2024 article cited above.

Following publication, concerns were raised regarding the scientific validity of the article. An investigation was conducted in accordance with Frontiers’ policies.

It was found that the complaints were valid and that the article does not meet the standards of editorial and scientific soundness for Frontiers in Endocrinology; therefore, the article has been retracted.

This retraction was approved by the Chief Editors of Frontiers in Endocrinology and the Chief Executive Editor of Frontiers. The authors did not provide a response to this Retraction.

